# Clinical characteristics of rheumatic disease-associated hypophysitis: A case series and review of literature

**DOI:** 10.1097/MD.0000000000031338

**Published:** 2022-10-28

**Authors:** Rui Yan, Yue-Bo Jin, Xue-Rong Li, Liang Luo, Xiao-Min Liu, Jing He

**Affiliations:** a Department of Rheumatology and Immunology, Beijing Shunyi Hospital, Beijing, China; b Department of Rheumatology and Immunology, Beijing Key Laboratory for Rheumatism and Immune Diagnosis, Peking University People’s Hospital, Beijing, China; c Department of Rheumatology and Immunology, Chongqing University Three Gorges Hospital, Chongqing, China; d Department of Chinese Medical, Chongqing Yubei People’s Hospital, Chongqing, China.

**Keywords:** clinical characteristics, hypophysitis, rheumatic disease

## Abstract

Rheumatic diseases have been reported to sometimes involve the pituitary gland. This study aims to characterize the clinical features and outcomes of patients with rheumatic disease-associated hypophysitis. We used the electronic medical record system in our hospital to identify nine patients with pituitary involvement in rheumatoid disease. We summarized the clinical characteristics, radiographic findings, treatments, and clinical outcomes of the 9 patients. We also performed a systematic literature review of systemic lupus erythematosus (SLE) cases with pituitary involvement published in PubMed and Wanfang databases from 1995 to 2021, and eight patients with complete information were selected. In the nine-patient cohort, the median age was 54 years, and the spectrum of rheumatic diseases included immunoglobulin G4-related disease (IgG4RD) (4/9), SLE (2/9), vasculitis (2/9), and Sjögren syndrome (SS) (1/9). All patients had pituitary abnormalities on radiological assessment, 6 developed diabetes insipidus (DI), and 8 presented with anterior pituitary hormone deficiencies in the disease duration. All the patients had multisystem involvement. As compared to hypophysitis with IgG4RD (IgG4-H), the age at onset of hypophysitis with SLE (SLE-H) patients was younger [(30.4 ± 16.4) years vs. (56.0 ± 0.8) years] and the disease duration was shorter [(14.0 ± 17.5) months vs. (71.0 ± 60.9) months] (*P* < .05). All patients were managed with glucocorticoids (GC) in combination with another immunosuppressant, and the majority of patients improved within 4 months. Six patients achieved disease remission while four required at least one hormone replacement therapy. Hypophysitis is a rare complication secondary to a variety of various rheumatic diseases that can occur at any stage. GC combined with additional immunosuppressants could improve patients’ symptoms; however some patients also required long-term hormone replacement therapy in pituitary disorders.

## 1. Introduction

Autoimmune hypophysitis (AH) is a rare autoimmune inflammatory disease affecting the pituitary gland that can lead to pituitary dysfunction. The severity of disease experienced varies, with 3% of patients not requiring treatment and most others needing long-term hormone replacement therapy. Some patients with severe disease can experience adrenal crisis, which is potentially life-threatening.^[1]^ It has been reported in the literature that 20% to 56% of AH cases are associated with other autoimmune diseases or drugs, such as Graves’ disease, granulomatous polyangitis, Sjögren syndrome, IgG4-related diseases, as well as a side effect related to immune checkpoint inhibitors, which have only recently gained attention.^[2]^ However, the incidence rate is low, with most cases being documented in case reports. We analyzed the clinical and immunological characteristics, treatment, and prognosis of nine patients with rheumatic disease-related hypophysitis to improve clinicians’ understanding of this disease. Pituitary involvement needs to be recognized at an early stage to avoid unnecessary biopsies of sellar lesions encountered in the context of rheumatic disease, and to minimize the risk of irreversible pituitary function loss by prompt implementation of a defined treatment protocol.

## 2. Materials and Methods

### 2.1. Study subjects

This is a retrospective observational study carried out at the Department of Rheumatology and Immunology at Peking University People’s Hospital (Beijing, China) from June 2003 to March 2021. Nine patients with clinically confirmed pituitary involvement formed the basis of this study. Clinical data, including disease manifestations, laboratory investigations, magnetic resonance imaging manifestations, concurrent diseases, therapy, clinical courses and outcomes, were retrospectively reviewed and recorded from case notes. The diagnostic criteria for hypophysitis were proposed by clinical guidelines of the Japan Endocrine Society and included the following: combined or secondary to established rheumatic disease; surgical or biopsy specimens showed lymphocytic hypophysitis by pathology; for patients without biopsy, diagnosis was based on clinical symptoms, biochemical examination, pituitary MRI findings, and effective immunosuppressive therapy, and excluding other pituitary diseases, such as pituitary adenoma, craniopharyngioma, germinoma, and other pituitary diseases localized to the sellar region. The diagnosis could be established if case criteria 1 + 2 or 1 + 3 were fulfilled.^[3]^ The diagnosis of all rheumatic diseases met the corresponding classification diagnostic criteria in this field. Meanwhile, we searched Pubmed and Wanfang database using the terms “SLE and hypophysitis, pituitary dysfunction, pituitary insufficiency, pituitary abnormality, pituitary enlargement, or diabetes insipidus” from 1995 to 2021, and eight patients with complete information were selected.^[4–11]^ The eight patients and our two patients made a group of ten patients of SLE-H, which we compared with our four hypophysitis with IgG4RD (IgG4-H). All patients on GCs and other immunosuppressants signed informed consents; an ethics committee was not needed for such retrospective analysis.

### 2.2. Statistical analysis

Normally distributed variables were described using arithmetic means and standard deviations (SD). Median and interquartile ranges were used for those quantitative nonparametrical variables. To analyze differences across hypophysitis subtypes, we performed one-way ANOVA or Kruskal–Wallis analyses. All statistical analyses were performed by SPSS version 24.0 (SPSS, Chicago, IL, USA) and GraphPad Prism software version 7.0 (Graph-Pad, San Diego, CA, USA). *P*-value < .05 was considered statistically significant.

## 3. Results

### 3.1. Patients’ characteristics

**Figure 1.**
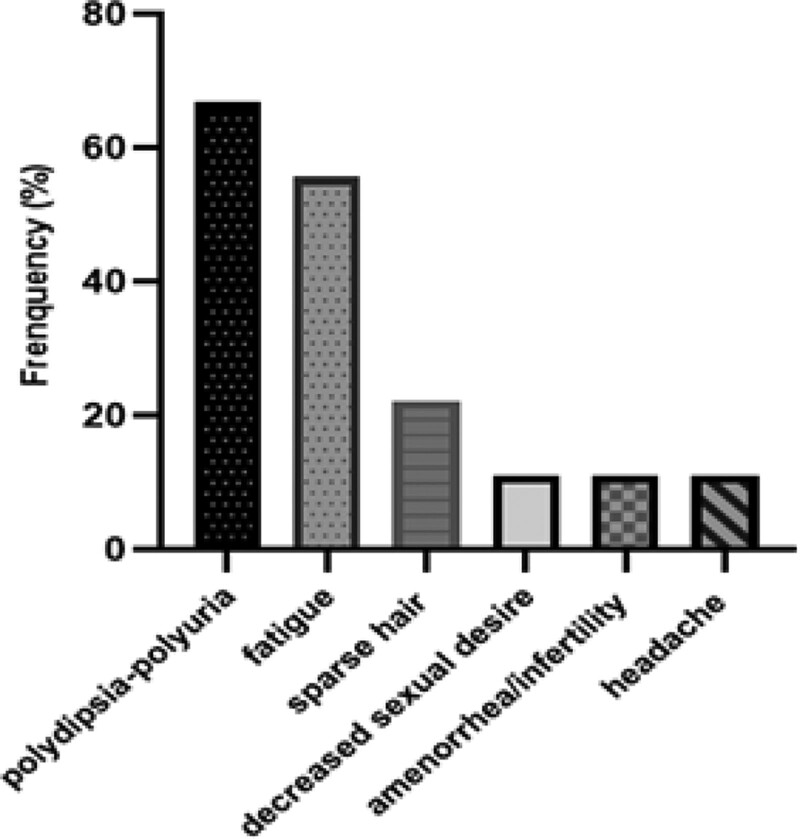
Clinical manifestations of hypophysitis of our cases.

Our cases included five men and four women, and the median age at diagnosis was 54 years old (ranged from 35 to 64). Clinical manifestations of hypophysitis were shown in Figure 1. One patient (case 3) had been mistaken for pituitary adenoma for two years and was operated on twice for compression caused by enlargement of the pituitary gland. Data for each patient was shown in Table 1.

**Table 1:** Patients’ characteristics.

Case no		1	2	3	4	5	6	7	8	9
Sex		M	M	F	F	M	F	M	M	F
Age (years)		64	55	35	35	56	56	58	57	52
Associated disease		Systemic vasculitis	IgG4RD	SLE	SS	IgG4RD	IgG4RD	EGPA	IgG4RD	SLE + SS
Disease duration (months)		3	36	108	5	4	144	36	96	24
Systemic extrapituitary manifestations		Acral pain, gangrene, oral ulcer	Dry mouth and eyes, submandibu-lar gland enlargement	Proteinuria	Fever, dry mouth, thirst	Abdominal pain, chest tightness	Swollen eyes, cough, chest tightness	Tinnitus, hearing loss, fever, cough	Chest tightness, lymph node enlargement, dry mouth and eyes	Dry mouth and eyes, echymosis, epistaxis, cough
*Extrapituitary involvement*		Skin, lung, small vessels	Submandib-ular gland, ureter, lung	Kidney, blood system	Kidney	Pancreas, bile duct, aorta	Eyes, lung, parotid gland, pancreas, kidney	Peripheral neuropathy, ENT, lung	Parotid gland, submandibular gland, lymph node, kidney	Lung, kidney, blood system
Hypophysitis duration(months)		12	3	108	0.1	4	1	4	96	24
Presentation on set		Polydipsia-polyuria, fatigue	Polydipsia-polyuria, fatigue, weakness	Polydipsia-polyuria, proteinuria	Polydipsia-polyuria,fatigue	Polydipsia-polyuria	Polydipsia-polyuria, fatigue	Headache	Sparse hair, hyposexuality	Sparse hair, Infertility, fatigue
Pituitary dysfuction	CDI	+	+	+	+	+	+	–	–	–
	FSH/LH	+	–/+	–	–	+	–	+	+	–
	TSH	–	–	–	–	+	–	+	–	+
	ACTH	–	+	–	+	+	+	–	+	+
	GH	–	–	–	–	–	–	–	–	–
	PRL	+	–	–	–	+	–	–	–	–
MRI findings		Pituitary enlargement,loss of posterior pituitary spot	Pituitary enlargement	Pituitary enlargeme-nt	Pituitary enlargeme-nt, loss of posterior pituitary spot	Stalk thickness	Stalk thickness, loss of posterior pituitary spot	Pituitary enlargeme-nt	Empty sella	Pituitary enlargement
Treatments		Pred+CYC+ADH	Pred+CYC+ADH	Pred+HCQ	Pred+MMF+ADH	Pred+CYC+ADH+ LTHCYC→RTX	Pred+RTX	Pred+RTX	Pred+CYC	Pred+IVIG+CsA+LTH
Follow up duration (months)		62	52	4	12	10	11	6	6	38
Outcome	Systemic extrapit-uitary status	Remission under treatment	Recurrence after 1 year of remission	Partial remission at 4 month follow-up, then lost	Partial remission	Remission under treatment	Remission under treatment	Remission under treatment	Remission under treatment	Remission under treatment
	Pituitary function	Recover	Partial remission with CDI		Recover	Persistent hypogonadism and CDI	Partial remission with CDI	Recover	Persistent hypogonadism	Recover

ACTH = adrenocorticotropin hormone, ADH = antidiuretic hormone, CDI = central diabetes insipidus, CsA = cyclosporine, CYC = cyclophosphamide, EGPA = eosinophilic granulomatous polyangitis, ENT = ear, nose and throat, FSH = follicle stimulating hormone, GH = growth hormone, HCQ = hydroxychloroquine, IgG4RD = immunoglobulin G4-related disease, IVIG = intravenous immunoglobulin, LH = luteinizing hormone, LTH = levothyroxine, MMF = mycophenolate mofetil, MRI = magnetic resonance image, Pred = prednisone, PRL = prolactin, RTX = rituximab, SLE = systemic lupus erythematosus, SS = Sjögren syndrome, TSH = thyroid stimulating hormone.

### 3.2. Evaluation of pituitary function

**Figure 2.**
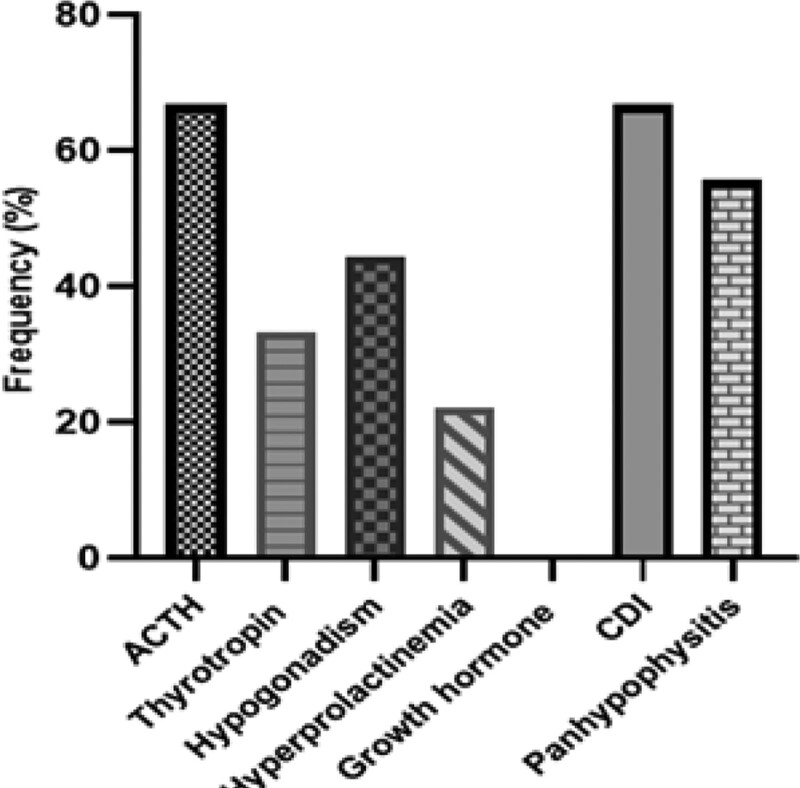
Manifestations of pituitary dysfunction of our cases.

In our cases, central diabetes insipidus (6/9) and adrenocorticotropin hormone (ACTH) insufficiency (6/9) were the most common hormone deficiencies experienced, followed by hypogonadism (4/9), thyrotropin (3/9), hyperprolactinemia (2/9), and no growth hormone (GH) deficiency (0/9). neurohypophysitis (6/9) and panhypophysitis (5/9) were found to be more prevalent than isolated anterior pituitary gland dysfunction (3/9) (Fig. 2).

### 3.3. Laboratory and imaging evaluation

**Figure 3.**
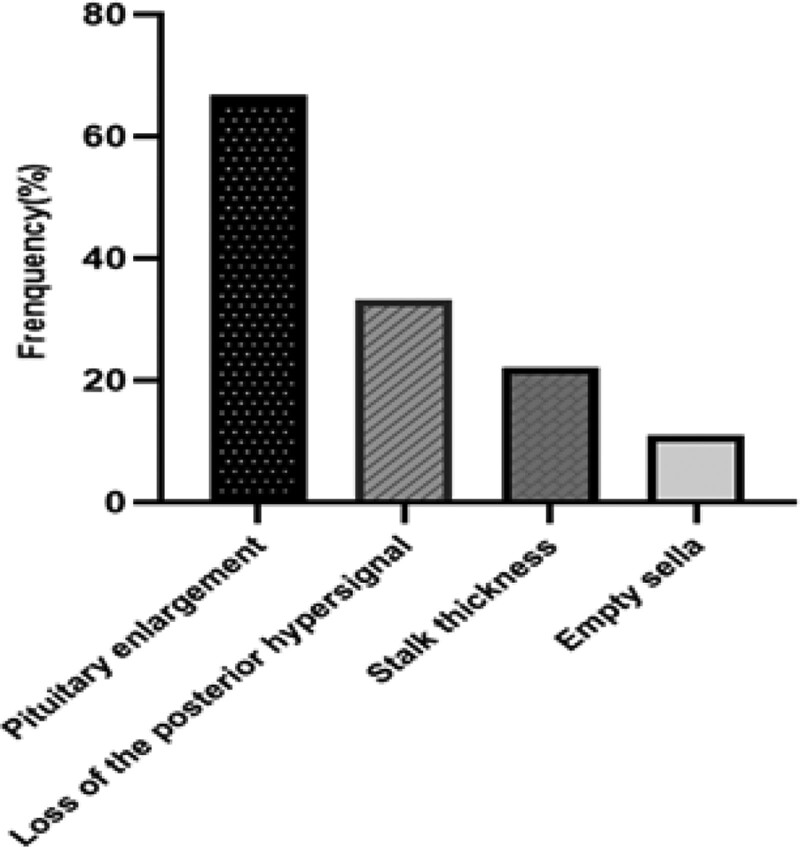
Radiological abnormalities of the pituitary of our cases.

Erythrocyte sedimentation rate (ESR) was not high in three patients and C reactive protein (CRP) was not high in two patients. All patients had abnormal pituitary images on MRI. Six of nine patients presented with pituitary enlargement (6/9), which was more than the loss of the posterior hypersignal (3/9) and stalk thickness (2/9) (Fig. 3).

### 3.4. Comparisons of hypophysitis characteristics in patients with systemic lupus erythematosus (SLE) and IgG4RD

As compared to IgG4-H, the age at onset of hypophysitis with SLE (SLE-H) patients was younger [(30.4 ± 16.4) years vs. (56.0 ± 0.8) years] and the disease duration was shorter [(14.0 ± 17.5) months vs. (71.0 ± 60.9) months, *P* < .05]. There were no differences in gender, pituitary gland axis, ESR, and CRP between the two groups. The comparisons between IgG4-H and SLE-H were shown in Table 2. The differences between SLE-non-H and SLE-H were shown in Table 3 and differences between IgG4H and IgG4-non-H were shown in Table 4.

**Table 2:** Comparison of characteristics between IgG4-H and SLE-H.

	IgG4-H (n = 4)	SLE-H (n = 10)	*P*
M/F ratio	3:1	3:7	>.05
Age on set(years)	56.0 ± 0.8	30.4 ± 16.4	<.05
Disease duration(months)	71.0 ± 60.9	14.0 ± 17.5	<.05
**Pituitary function**			
Corticotropin axis	4	3	>.05
Thyrotropin axis	1	6	>.05
Gonadotropin axis	2	6	>.05
Hyperprolactinemia	1	5	>.05
CDI	3	6	>.05
Panhypophysitis	3	5	>.05
**Radiology findings**			
Pituitary enlargement	1	7	>.05
Empty sellar	1	1	>.05
Loss of posterior pituitary spot	0	1	>.05
Stalk thickness	4	2	>.05
**Therapy**			
Dosage ofprednisone (mg)	30	50	>.05

CDI = central diabetes insipidus, IgG4-H = hypophysitis with IgG4RD, SLE-H = hypophysitis with SLE.

**Table 3:** Comparison of characteristics between IgG4-non-H and IgG4-H.

	IgG4RD-non-H (n = 10)	IgG4-H (n = 4)	*P*
Female	6	1	>.05
Male	4	3	>.05
Age (years)	53.4 ± 11.1	55.5 ± 0.6	>.05
Disease duration (months)	53.9 ± 43.7	70.0 ± 59.2	>.05
Eosinophilia (%)	1.8 ± 1.4	6.0 ± 4.0	<.05
IgE (IU/mL)	79.0 (9.1‐1432)	375.5 (164‐893)	<.05
IgG4 (mg/dL)	202.0 (24.5‐3980)	2642.5 (397‐7519)	<.05
ESR (mm/h)	10.4 ± 10.5	23.3 ± 25.5	>.05
CRP (mg/L)	1.5 ± 2.3	1.2 ± 1.4	>.05
Number of T lymphocytes	1652.9 ± 808.5	1869.7 ± 708.5	>.05
Number of B lymphocytes	223.1 ± 265.6	106.7 ± 19.1	>.05

IgG4-H = hypophysitis with IgG4RD.

**Table 4:** Comparison of characteristics between IgG4-H and SLE-H.

	IgG4-H (n = 4)	SLE-H (n = 10)	*P*
M/F ratio	3:1	3:7	>.05
Age on set (years)	56.0 ± 0.8	30.4 ± 16.4	<.05
Disease duration (months)	71.0 ± 60.9	14.0 ± 17.5	<.05
**Pituitary function**			
Corticotropin axis	4	3	>.05
Thyrotropin axis	1	6	>.05
Gonadotropin axis	2	6	>.05
Hyperprolactinemia	1	5	>.05
CDI	3	6	>.05
Panhypophysitis	3	5	>.05
**Radiology findings**			
Pituitary enlargement	1	7	>.05
Empty sellar	1	1	>.05
Loss of posterior pituitary spot	0	1	>.05
Stalk thickness	4	2	>.05
**Therapy**			
Dosage of prednisone (mg)	30	50	>.05

IgG4-H = hypophysitis with IgG4RD, SLE-H = hypophysitis with SLE.

### 3.5. Outcomes

**Figure 4.**
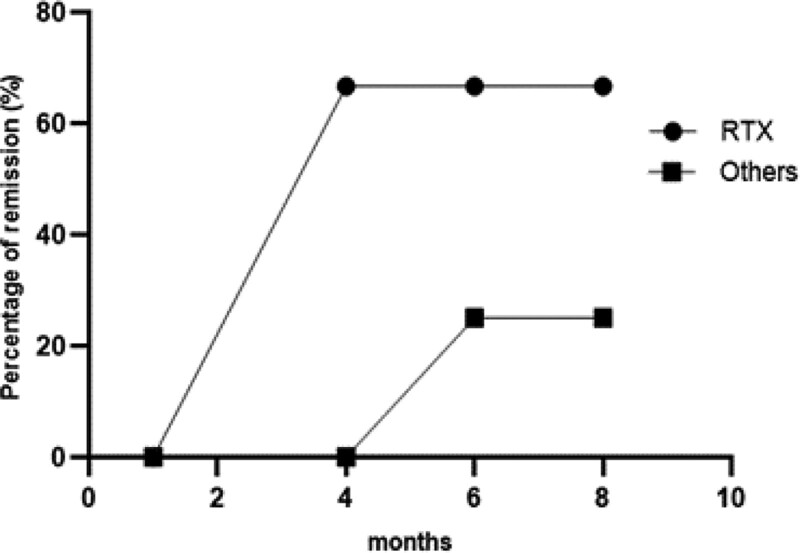
Patient remission over time of our cases.

All patients received GCs and other immunosuppressive agents, including cyclophosphamide (CYC) (4/9), hydroxychloroquine (1/9), cyclosporine (CsA) (1/9), and rituximab (RTX) (3/9). One SLE-H patient (case 9) received intravenous immunoglobulin in addition to GCs and CsA. One IgG4-H patient (case 5) was treated with GCs and CYC for half a year, and while their systemic symptoms improved, their posterior pituitary function was not improved, and then CYC was changed to RTX. Four of the nine patients accepted hormonal replacement therapy. At the end of follow-up (from 3 to 62 months), six patients achieved whole remission of systemic disease, while only four patients recovered pituitary function. Four patients required at least one hormonal replacement therapy. Comparison of treatment between RTX and other therapies was shown in Figure 4.

## 4. Discussion

In this study, we describe nine cases of pituitary involvement in rheumatic diseases from a single center during an 18-year period and summarized the clinical, laboratory, radiological, and imaging features and treatment response. Hypophysitis is a rare autoimmune disease and was first described by Goudie and Pinkerton in 1962.^[12]^ At first, it was thought that it mostly occurred during late pregnancy and out until 6 months postpartum. The ratio of male to female was 1:8. It is now known, however, that AH can occur in men and non-pregnant women. A statistical analysis of 76 cases of hypophysitis in Germany showed that only 11% of the cases were related to pregnancy.^[13]^ The ratio of male to female in this study was 5:4, but there were differences in the proportion of men and women in different diseases, such as 3:7 in SLE, 1:3 in IgG4RD, which seemed related to a small number of cases and none of them were related to pregnancy.

Da Silva^[14]^ reported that the incidence rate of SLE complicated with hypophysis was 1.3% and for Sjögren syndrome it was 0.8%. Pituitary involvement in granulomatosis with polyangiitis (GPA) was about 0.8% to 2%.^[15,16]^ In short, the incidence rate of hypophysis is not high in rheumatic diseases. In recent years, increased disease recognition thanks to the development of better imaging technology has allowed for more cases to be diagnosed. GPA and IgG4RD were the most frequent diseases associated with hypophysitis in the reported literature.

Hypophysitis can occur in various stages of rheumatic diseases and can also be the only or first manifestation of disease. Arturo reported GPA started with pituitary dysfunction in 46%, either with or without other systemic symptoms of vasculitis.^[17]^ Our study showed that the clinical manifestations of hypophysitis in one patient appeared 1 to 9 years before rheumatic diseases had been diagnosed, in four patients it coexisted with other system/organ involvement, and in four other patients it appeared 5 to 12 years after diagnosis of rheumatic disease, which reminds us that the manifestation of hypophysitis can be unpredictable and that we should pay more attention to patients’ medical histories and follow them closely to make early diagnoses. A comparison of cumulative characteristics of patients with secondary/complicated hypophysitis from previous reports and our cases can be seen in Table 5. In our cases, the age of SLE-H onset was younger than IgG4H, and we speculated that this was related to the age of the disease prone population. SLE is more common in young women, while IgG4RD is more common in middle-aged and elderly men.

**Table 5:** Cumulative characteristics of patients with secondary/complicated with hypophysitis in previous reports.

	Our series	Kapoor^[15]^	Liu^[16]^	Arturo^[17]^	Shikuma^[18]^	Li^[19]^	Li^[20]^
Established rheumatic disease		GPA	GPA	GPA	IgG4RD	IgG4RD	IgG4RD
Case no	9	8	66	74	84	5	76
Male/female ratio	5:4	1:1	20:46	1:2	2.4:1	3:2	1.5:1
Age at hypophysitis involvement (years)	54	48	NR	43 ± 15	64.2 ± 13.9	49.6 ± 2.9	54.1 ± 17.8
Clinical features (n, %)							
Headache	1 (11.1)	1 (12.5)	NR	8 (12)	NR	0 (0)	20 (26.3)
Corticotropin axis	6 (66.7)	1 (12.5)	14 (21.2)	19 (28)	39 (46.4)	3 (60)	48 (63.2)
Thyrotropin axis	3 (33.3)	4 (50)	27 (40.9)	25 (37)	34 (40.5)	4 (80)	45 (59.2)
Gonadotropin axis	4 (44.4)	7 (87.5)	34 (51.5)	38 (53)	40 (47.6)	3 (60)	52 (68.4)
Hyperprolactinemia	2 (22.2)	1 (12.5)	19 (28.8)	25 (37)	NR	NR	32 (42.1)
Growth hormone axis	0 (0)	1 (12.5)	7 (10.6)	9 (15)	34 (40.5)	4 (80)	37 (48.7)
CDI	6 (66.7)	6 (75)	57 (86.4)	65 (88)	59 (70.2)	4 (80)	30 (39.5)
Panhypophysitis	5 (55.6)	3 (37.5)	NR	17(23)	44 (52.4)	2 (40)	44 (57.9)
Radiology findings (n, %)							
Pituitary enlargement/sellar mass	5 (55.6)	6 (75.0)	49 (74.2)	NR	12 (14.3)	NR	39 (51.3)
Empty sellar	1 (11.1)	1 (12.5)	NR	NR	NR	1 (20)	NR
Loss of posterior pituitary spot	3 (33.3)	NR	25 (37.9)	28 (46)	NR	1 (20)	NR
Stalk thickness	2 (22.2)	NR	12 (18.2)	29 (41)	18 (21.4)	4 (80)	20 (26.3)
Outcomes (%)							
Glucocorticoid	100	100	79	75	85	NR	NR
Remission	78	67	70	79.4	97	NR	94

It had been reported that hypophysitis can occur in associated with SLE, antiphospholipid syndrome (APS), GPA, IgG4RD, etc,^[21]^ but the mechanism of rheumatic disease-associated hypophysitis has not been made clear. Lymphocytic hypophysitis (LH) is the most common subtype of AH.^[22]^ The pathological mechanism behind AH is the infiltration of T and B lymphocytes into the pituitary gland, where they form lymphoid follicles with germinal centers. Different degrees of fibrosis can occur in different areas of the pituitary gland, and plasma cells and macrophages can also infiltrate, which varies depending on the specific associated autoimmune disease.^[23]^ It is well known that vasculitis is the pathological basis of SLE, while IgG4RD is characterized by infiltration of IgG4-positive plasma cells into various organs. In our study, the disease duration of SLE-H was shorter than IgG4-H. This may indicate that vasculitis occurs earlier than granulomatous lesions in hypophysitis. No biopsies, however, was performed in our cases and we need a larger sample to make an accurate judgment. In fact, it is currently difficult to perform pituitary biopsy in clinical work, so clinicians should develop noninvasive diagnostic techniques based on clinical manifestations and imaging characteristics.

The Mayo Clinic reported 637 patients with GPA from 1996 to 2011 and thought that neither the Birmingham vasculitis score nor the GPA injury index could be used to assess the occurrence and severity of GPA-H.^[15]^ In this study, ESR was not high in three of the nine patients, and CRP was not high in two of the nine patients. These findings may prompt us to conclude that hypophysitis is not completely parallel in disease activity across different associated conditions. Further clinical studies with larger sample sizes are warranted.

Due to the heterogeneity of clinical manifestations, it is important to distinguish between the acute phase of hypophysitis, which may require systemic treatment, and the chronic phase, which often evolves into fibrosis and pituitary atrophy, and only treatment of hypopituitarism is needed.^[24]^ The guidelines recommended by the Japanese Endocrine Society emphasize that treatment of IgG4-H should follow the recommendations of treating IgG4RD.^[25]^ GCs are recommended as a first-line drug for the treatment of IgG4-H. GCs can reduce pituitary hyperplasia and pituitary stalk thickening. In addition, azathioprine, methotrexate, and cyclosporine have also been reported to be effective. Rituximab has potential benefits in the treatment of refractory or recurrent hypophysitis, which has been reported to occur in IgG4-H and GPA-H, especially those with B lymphocyte predominance. In our series, all patients were treated by GCs combined with other immunosuppressants. Among them, three patients were treated with prednisone and RTX, and four patients were treated with prednisone and CYC. One IgG4-H patient was treated with GCs and CYC for half a year, systemic symptoms were improved, but function of the posterior pituitary gland was not improved, and then CYC was changed over to RTX. Due to a limited number of cases, there is a lack of clinical trials comparing the efficacy of different therapeutic strategies. Whether RTX can be of benefit for AH patients needs to be further studied.

## 5. Conclusion

Hypophysitis is a rare complication secondary to a variety of rheumatic diseases. GCs combined with immunosuppressants can improve the patients’ symptoms, but some patients require additional long-term hormone replacement therapy in pituitary disorders.

## Authors contributions

All authors analyzed and interpreted the patient’s data; Rui Yan and Yuebo Jin were major contributions in writing the manuscript. All authors read and approved the final manuscript.

**Conceptualization:** Jing He, Xue-Rong Li.

**Data curation:** Xiao-Min Liu.

**Formal analysis:** Xiao-Min Liu, Xue-Rong Li, Yue-Bo Jin

**Funding acquisition:** Xiao-Min Liu.

**Investigation:** Xue-Rong Li.

**Methodology:** Liang Luo, Rui Yan.

**Writing – original draft:** Rui Yan.

**Writing – review &amp; editing:** Jing He, Liang Luo, Rui Yan, Yue-Bo Jin.
